# Dismantling ableism in health professions education: imperative in psychosocial management of pain

**DOI:** 10.3389/fpubh.2026.1896570

**Published:** 2026-07-27

**Authors:** Krista F. Van Der Laan, Antoinette L. Spector

**Affiliations:** Department of Physical Therapy and Human Movement Sciences, Feinberg School of Medicine, Northwestern University, Chicago, IL, United States

**Keywords:** ableism, anti-ableism, biopsychosocial (BPS) model, chronic pain, disability inclusion, health professions education, hidden curriculum, pain management

## Abstract

Pain and chronic pain are prevalent and costly. Integrative models of pain management including the biopsychosocial (BPS) model of pain are central to contemporary public health efforts to address pain, however, uptake of the BPS model of pain in patient care is inconsistent. Aspects of health professions education (HPE) have been identified as barriers to BPS use including emphasis on the medical model and the hidden curriculum. Similarly, hidden curriculum in HPE limits disability inclusion and reduces healthcare accessibility. This perspective offers a conceptual framing of ableism as a driving force for both marginalizing and exclusionary features in HPE. Ableism centers on normative ways of functioning, thinking, and being, which contributes to training of providers who may not apply holistic and multidimensional understanding of pain. Dismantling ableism and working toward anti-ableist HPE requires recognition of ableism, cultivating environments that model anti-ableist approaches, and addressing the contributing systems. By transforming learning environments and promoting anti-ableist HPE, the same dispositional and structural conditions needed for BPS approaches to pain can be achieved.

## 1 Introduction

Chronic pain is among the most prevalent and costly health conditions in the United States. In 2023, an estimated 24.3% of U.S. adults (approximately 60 million people) reported chronic pain in the prior 3 months, and 8.5% reported high-impact chronic pain (HICP) — pain that limits life or work activities on most or every day (1). Chronic pain is one of the most common reasons adults seek medical care and is associated with reduced quality of life, lost productivity, increased anxiety and depression, harmful opioid use, and unmet mental health needs (2). By definition, HICP meets the Americans with Disabilities Act (3) criterion of “a physical or mental impairment that substantially limits one or more major life activities,” including walking, standing, lifting, sleeping, concentrating, and working. In healthcare, estimates of pain and work-related musculoskeletal disorders vary widely: 61% of healthcare workers report low back pain and 64% report hip pain (4); 77.2% of nurses report pain in various regions (5); 40.1% of physical therapists report low back pain (6); and 77% of orthopedic surgeons report low back pain and 74% report neck pain (7). Pain and HICP thus carry significant population-level relevance for the health professions workforce, with implications for how clinicians are trained and prepared to deliver equitable care.

Use of the biopsychosocial (BPS) model (8) has been emphasized in the examination and treatment of people with pain (9). The BPS model of pain conceptualizes pain as a multidimensional experience shaped by the dynamic interaction of biological (e.g., tissue pathology, nociception, genetics), psychological (e.g., cognitions, emotions, behaviors), and social (e.g., relationships, culture, environment, socioeconomic context) factors (8, 9). While biological aspects of pain have historically been the predominant focus, health professions education (HPE) programs now integrate more psychosocial content within formal coursework (10–14). Similarly, clinical care for pain reflects improvements in taking an integrative approach (15). In this manuscript, integrative pain management refers to a coordinated, person-centered approach that combines multiple evidence-informed strategies to address biopsychosocial contributors to a person’s pain (16).

Despite advances, barriers to BPS implementation have been identified. A scoping review by van Dijk et al. (15) identified categories of barriers including: (1) clinician: inclination toward biomedical contributions, negatively framing the presence of psychological and social factors, and lack of mentoring in BPS; (2) patient: treatment expectations; and (3) system: inadequate time and decreased knowledge of referral sources. Elements within training environments of HCP create additional barriers to acceptance and integration of psychosocial management of pain in clinical practice (15–18). The “hidden curriculum” has been identified as a contributing factor to the delay in the translation of the BPS to clinical practice (18). Hidden curricula are those aspects of an educational program that are implicit within attitudes of educators and policies of a program (18–20) (Table 1). The learning effects from hidden curricula are often longer lasting than aspects of the formal or explicit curricula in HPE (21).

**Table 1:** Key terminology.

Term	Definition
Hidden curriculum	The implicit attitudes, norms, and policies in training environments that shape learners’ beliefs and behaviors but are not formally taught.
Haunted curriculum	Aspects of health professions education shaped by enduring “violent histories” (e.g., racism, sexism, ageism, ableism) that continue to permeate the present (21)
Ableism	A system that assigns value to bodies and minds based on norms of normalcy, productivity, and fitness, resulting in prejudice and exclusion of disabled people (25, 26).
Capability imperative	The implicit expectation that health professionals be “superhuman,” showing no weakness or needs, and maintaining peak performance at all times (45).
Students with disabilities (SWD)	Learners who experience disability, including pain-related disability, and who may require accommodation or structural changes to participate fully in training.
Anti-ableist health professions education	Educational structures and practices that intentionally identify, disrupt, and redesign policies, curricula, and cultures that marginalize disabled learners and clinicians.
Universal Design for Learning (UDL)	An educational framework that emphasizes flexible methods, materials, and assessments to support engagement and success for diverse learners (66).

A more recent concept in HPE is that of the “haunted” curriculum (22). Ansari et al. (22) define the haunted curriculum as, “an evocative conceptual framing to engage with aspects of the medical and HPE that are toxic and troubling to learners and to understand how violent histories loom large and continue to permeate the present.” (p. 1) They derived the concept from work done by Viviane Saleh-Hanna (23) on hauntology in Black-feminism. The haunted curriculum in the training environments of HPE include the effects of racism, sexism, ageism, and ableism.

Ableism as part of the haunted curriculum creates a normalized body/mind (24), driving a focus on the biomedical understanding of human ability (25) and reducing inclusion of persons with disabilities (PWD) in health professions. Ableism is defined as “a system of assigning value to people’s bodies and minds based on societally constructed ideas of normalcy, productivity, desirability, intelligence, excellence, and fitness” (26) that results in “discrimination of and social prejudice against people with disabilities. At its heart, ableism is rooted in the assumption that disabled people require ‘fixing’ and defines people by their disability” (27). Ableism appears in multiple ways within clinical care. Relative to physical environments, clinical spaces lack adaptations to the needs of PWD (e.g., fixed height exam tables preventing adaptations to patients’ function). Additionally, clinicians claim they cannot modify procedures to different bodies (28, 29), hold biases about disability in general (29, 30), emphasize the biomedical understanding in all conditions including those with pain (15), and view conditions that do not have an apparent biological driver as overly complex or attributable to difficult patients (18, 25, 31). Additionally HCP with visible or disclosed disabilities are often absent from the workforce (32). In academic and training environments, ableism appears with programs centering on students with normative needs and expressed through emphasizing the medical model of disability (i.e., disability is an individual problem, biological in nature, and needs to be fixed) (20).

When clinical and training environments reinforce a singular way of being that excludes patients and learners outside a hegemonic ideal, it follows logically that we cannot set the groundwork for uptake of holistic and multi-dimensional approaches inherent in the BPS model. Addressing ableism in health care requires acknowledging its impact in HPE and actively creating anti-ableist training environments. In this perspective, we offer a conceptual framing of barriers ableism creates for full integration of the BPS model of pain and highlight specific actions to dismantle, and thereby reduce the influence of, ableism.

## 2 Ableism from hidden to “haunted”

The concept of the hidden curriculum was introduced in medical education by Frederic Hafferty and Ronald Franks (19). Mardian et al. (18) highlight the need to address the hidden curriculum surrounding psychosocial management of pain, though reconceptualizing the BPS model to the “sociopsychobiological” framework. They suggest that, despite international recommendations for BPS use, learners observe instructors emphasize biophysiological causes for pain or prescribe treatments aimed at the biomedical without considering nor screening for other dimensions. Overlapping that, the hidden curriculum promotes exclusion of individuals with disability. Examples include technical standards (33), program policies (34), fitness for duty assessments (33), deep-seated beliefs about disability (29, 35) and who may enter the health professions (20, 36). Repairing and reducing the hidden curriculum both in what is communicated about people with pain and/or disabilities and who may enter the health professions are vital steps but may not go far enough to fully address systemic barriers and advance care for people with pain.

What has not been described in the pain literature, and we submit as the genesis for above mentioned hidden curriculum, are the effects of ableism as part of the “haunted curriculum” in HPE (22). ^“^Haunted” aspects of training in HPE continue to perpetuate marginalization and exclusion of certain groups of trainees and HCP and fuel hidden curricula. Ableism, as part of the haunted curriculum (24), is a historical feature of healthcare and HPE. Healthcare has had a fraught relationship with disability including the eugenics movement (37) and emphasis on the medical model of disability (37–40). Both imply the need to remove and/or to fix disability rather than understand the social determinants and normalize it as a regular feature of human existence (41). Work by Vanpuymbrouck et al. (35) highlights the frequent tendencies to aversive ableism in physical therapists and occupational therapists. Aversive ableism is an implicit bias where outwardly people demonstrate positive views of PWD but internally demonstrate a preference toward non-disabled people (42). While aversive ableism is not explicitly ableist, and the professions of physical and occupational therapy often promote disability inclusion, features in the training and professional environments of these and other health professions perpetuate ableism.

Students with disabilities (SWD) encounter ableism-fueled barriers for entry and completion of degrees (32, 34, 43–46). They must simultaneously manage the roles of patient and professional by considering what about their disability to disclose, managing their health condition, requesting accommodations versus using flexibility inherent in the environment, and using their personal healthcare experiences in the service of patients (45, 46). Additionally, low numbers of individuals with apparent disabilities in the health professions reduces representation, increases isolation, and results in environments built for hegemonic learning needs (32). The result is less PWD, including those with HICP, who appear in academic spaces and progress to practice clinically, providing continued support for ableism to persist.

Jain (47) identifies the “capability imperative” as a driver of ableism in HPE. The capability imperative is an implied message reinforced to healthcare trainees that they must be “superhuman,” where they must “show no weakness [and] have no needs,” maintaining peak physical condition justified by altruistic service to patients. SWD are often not believed when they request what they need, and their accommodations are frequently denied, being perceived as unreasonable (20, 48). Students who successfully complete training are those who meet or adjust to hegemonic expectations, policies and structures rather than representative of society. This feels eerily similar to the experience of people with HICP, having to overly justify symptoms to providers who either entirely attach the condition to ‘problematic patients’ or dismiss how psychological and social factors interact with the biomedical (49–52).

**Figure 1:**
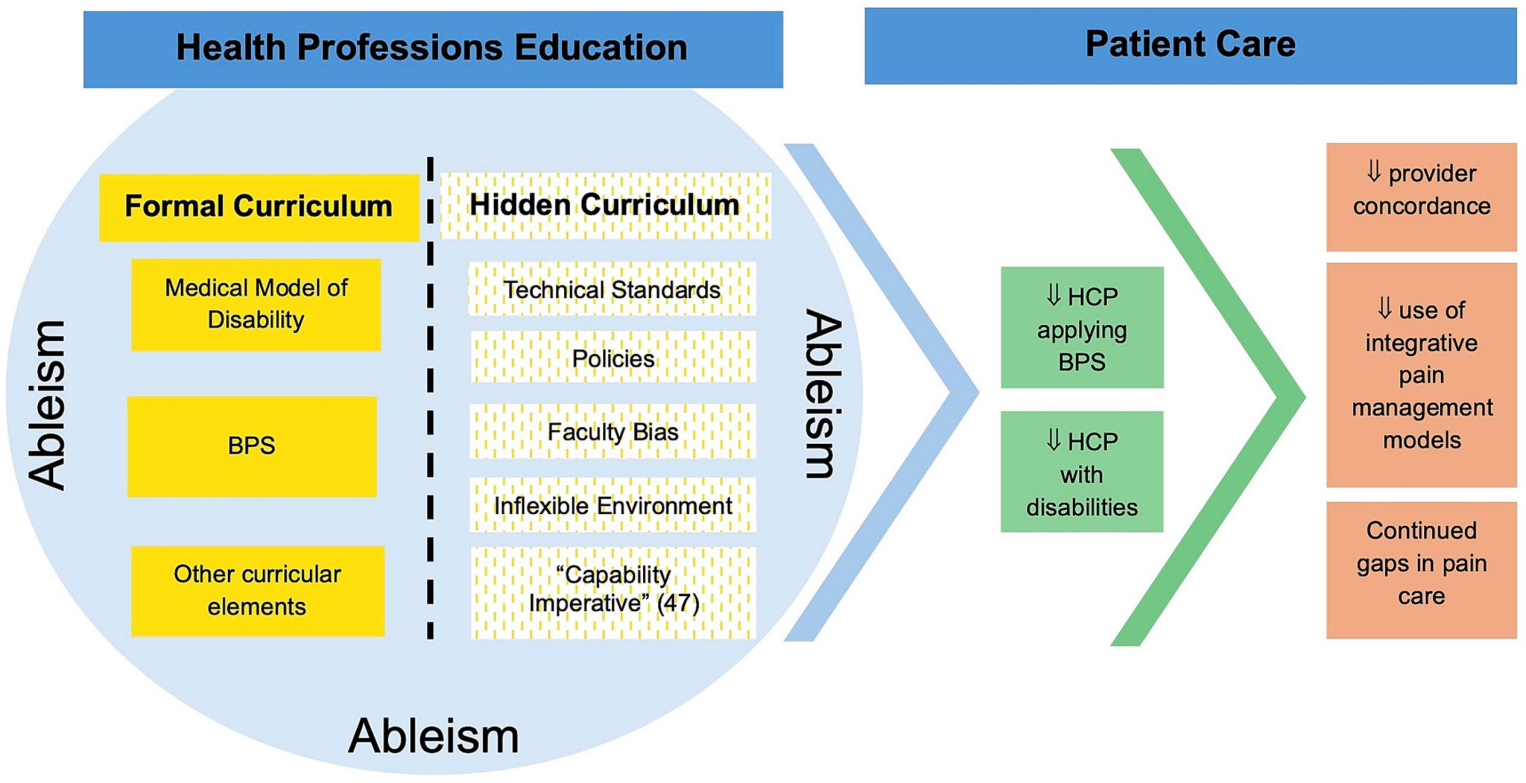
Conceptual framework of ableism’s effects on HPE and integration of BPS model of pain. Conceptual framing of ableism as a driver of aspects of HPE that reduce application of the BPS model of pain in patient care. Suggested effects are the provider level and directly with the patient, possibly resulting in continued gaps in pain care. HPE, Health Professional Education; BPS, biopsychosocial mode of pain management; HCP, Healthcare Professional. 47. Jain N. The capability imperative: theorizing ableism in medical education. Soc Sci Med. (2022) 315:115549. doi:10.1016/j.socscimed.2022.115549.

Ableism drives the focus on an ideal body/mind and notions of “fixable” conditions, where pain improves with solely biomedically directed interventions, and it limits HPE from being an inclusive space for people with disabilities. Until we address ableism in healthcare learning spaces, we will likely continue to have graduates with an intellectual understanding of the BPS model of pain, but with a disconnect of what looks like in its most actualized form (Figure 1).

## 3 Dismantling ableism

### 3.1 Acknowledging ableism

Recognizing ableism and actively creating an anti-ableist environment are foundational steps towards reducing its role in HPE. Uncovering biases first requires vulnerability to acknowledge other’s experiences despite no first-hand knowledge ourselves (53, 54). Vulnerability also allows us to explore and see the reality of the environment of HPE and how it perpetuates ableism as Nicole Piemonte (54) highlights in her book *Afflicted: How vulnerability can heal medical education and practice*. She writes that we need learning environments that explicitly demonstrate that, “because we all share the potential to suffer, there is very little that separates doctors from patients” (54, p. 168). All bodies and minds have the potential for ability and disability, and an anti-ableist environment does not elevate ideal ways to exist.

Misbeliefs abound related to SWD and providers with disabilities. Myths that specifically disadvantage SWD include concerns of safety, altering professional standards, and costly accommodations (35, 55). These concerns of safety have been unfounded (55). Without acknowledging ableism’s role in the current learning and clinical environments, myths will prevail and the disclosure of disability and the use of necessary accommodations will continue to have career limiting effects.

A pain-related example demonstrating the necessity of accommodations for disability is a requirement of working long hours without a break, which could be untenable for someone with HICP. That trainee would then either opt out of the learning experience, which may not be optional, or go through excessive steps to secure accommodations that ultimately might be denied. This person may not successfully complete training not due to their ability to provide effective care but due to an entirely modifiable environmental barrier (56). It’s possible that they fail to enter the workforce, though they may have the necessary knowledge, skills, and relevant lived experiences to be an excellent HCP. Provider concordance enhances care and improves patient outcomes in various populations (57) including for PWD (32, 43, 58). Limited literature suggests that personal or proximal experience of pain may improve understanding of BPS model of pain (59, 60). HCP with HICP may more easily enter and remain in the health professions workforce if training and practice environments were specifically anti-ableist and inclusive (61).

### 3.2 Modeling a psychosocial approach

Expansive uptake of the BPS model might also be increased if learners have a psychosocial approach modeled for them. As Englander et al. (62) identify, a HCP’s practice is directly influenced by their training environments. This goes beyond what Mardian et al. (18) highlight as limits in the hidden curriculum, which focuses on implicit messaging about treatment of patients in pain. Modeling a psychosocial approach directly to learners offers specific messaging about how learners should care for patients once they become HCP. The necessity of learner centered approaches is particularly salient for students with all types of disabilities including HICP. Psychosocially and context-informed relationships are modeled in educational environments that promote kindness as an essential metacognitive skill (63), broad inclusion, and anti-ableism. Learners gain experiential understanding of applying psychosocial approaches to the examination and treatment of people with pain (64) as they are the recipients and/or observers of a situated relationship that considers psychological, social, and contextual needs through a caring lens (54, 62). Modeling can result in an “empathic hidden curriculum” that emphasizes inclusion, wellbeing for HCP, and use of the BPS model in practice (64).

Educational frameworks can assist faculty with integrating and modeling psychosocial principles in anti-ableist HPE. Theall et al. (65) offer a conceptional framework of disability inclusion in medical education, illustrating the multi-dimensional levels in an environment that supports all students well, including SWD. Analogous to the BPS, it highlights material needs for the learning environment as well as social and personal needs promoting student success. Additional learning frameworks like Universal Design for Learning (66) offer methods for reaching greater numbers of learners in educational spaces, emphasizing the importance and foundational nature of creating a responsive environment.

### 3.3 Changing the systems

Disability scholars in HPE offer strategies to actively work against ableism to create anti-ableist education. While the following strategies are not specifically focused on pain-related disability, they broadly create anti-ableist environments, which would include those with HICP:

- Cocreation of content in educational settings with people who have pain, highlighting lived experience (41, 67, 68) and deemphasizing the medical model of disability by highlighting more holistic models of disability and pain (40, 69).
- Creating systems where PWD, including due to pain, can enter HPE alongside non-disabled peers (21, 70).
- Professional associations can champion efforts by offering specific supports for SWD and HCP with disabilities (33, 71). This includes guiding training programs to staff disability resource providers with expertise in creating inclusive classroom and clinical learning environments, facilitating entry into practice (70).
- Actively acknowledging the “capability imperative” (47) in HPE and clinical environments and eradicating it. This may include critical evaluation of “professionalism” standards, which can pressure trainees to meet unrealistic expectations and disregard normal human variability and needs (48, 70).
- Developing faculty to support anti-ableist HPE in all aspects of program curricula (20, 33, 41).
- Changing standards from accrediting bodies to support full inclusion of SWD (33, 70) and address “disability competence” (71) as an integrated feature of training HCP (41).

These tenable suggestions create opportunities to address ableism’s role in HPE and create inclusive environments where the BPS model is naturally promoted. They require foundations of vulnerability, recognition of ableism, modeling anti-ableist behaviors, and addressing systems that perpetuate ableism.

## 4 Discussion

Framing HPE as explicitly anti-ableist directly supports the integrative models of pain care called for in contemporary public health practice. Integrative pain management requires clinicians to move beyond a biomedical, deficit-oriented framework to focus on the biological, psychological, social, and contextual dimensions of pain. Anti-ableist HPE cultivates the same dispositional and structural conditions this work requires by normalizing variation in bodies and minds, modelling compassionate and curious inquiry, and treating accommodation, flexibility, and lived experience as legitimate sources of knowledge rather than as deviations from a normative standard. In doing so, it equips trainees to recognize pain as a complex, dynamic experience shaped by social and structural determinants, to engage patients as partners in recovery, and to draw on a broader repertoire of non-pharmacologic, resilience-building, and community-oriented strategies. Anti-ableist HPE can then be a driving force which shapes the workforce, attitudes, and practice environments through which integrative approaches can be reliably delivered at the population level.

Calls to expand disability inclusion in HPE frequently surface concerns about patient safety, clinical competency, and the integrity of professional competency. These concerns are most often raised in fields with physically demanding clinical requirements, such as physical therapy. Concerns – like the realities of manual patient handling and long clinical shifts – deserve direct engagement, not dismissal. Patient safety is a non-negotiable professional obligation, and programs bear genuine responsibility for ensuring that graduates can practice competently across the range of settings in which they will be licensed. Yet, they rest on assumptions the evidence does not support (35, 55). Technical standards in many programs were written decades ago and often reflect historical norms rather than current evidence about what clinical practice actually requires, functioning more as proxies for ableism than as valid measures of professional capability (36, 44, 72, 73). For example, a competency of safe patient handling could be demonstrated by a trainee personally lifting a patient of a specified weight, or through directing a transfer, using a mechanical lift, or supervising personnel. These patient handling methods are all routine in contemporary clinical practice and align with the competency being met versus specifying the pathway in which the standard must be met. With appropriate accommodations, trainees and clinicians with disabilities can not only meet performance expectations, but are also positioned to contribute distinctive clinical strengths, including stronger communication, patient rapport, and disability-competent care (32, 71). Reasonable accommodation is legally obligated under the ADA, consistent with HCPs’ responsibility to deliver safe, equitable care. Anti-ableist HPE does not relax standards. Instead, it asks programs to interrogate which standards genuinely safeguard patients and which simply preserve the status quo of who is permitted to become a clinician.

The full incorporation of the BPS model in pain management is long overdue. Many efforts have been made to translate what has been known for decades into clinical practice. Ableism, particularly in training environments, appears as a barrier to full integration due to its pervasive effects perpetuating a biomedical focus, exclusion, and marginalization (20, 21, 56). HPE can make the systemic changes needed to create anti-ableist environments supporting development of HCP who provide multidimensional and integrative care for all people.

## Data Availability

The original contributions presented in the study are included in the article/supplementary material, further inquiries can be directed to the corresponding author.
